# In Vivo Efficacy and Safety Evaluations of Therapeutic Splicing Correction Using U1 snRNA in the Mouse Retina

**DOI:** 10.3390/cells12060955

**Published:** 2023-03-21

**Authors:** Sebastian Swirski, Oliver May, Malte Ahlers, Bernd Wissinger, Martin Greschner, Christoph Jüschke, John Neidhardt

**Affiliations:** 1 Human Genetics, Department of Human Medicine, Faculty of Medicine and Health Sciences, University of Oldenburg, Carl-von-Ossietzky-Straße 9-11, 26129 Oldenburg, Germany; 2 Visual Neuroscience, Department of Neuroscience, Faculty of Medicine and Health Sciences, University of Oldenburg, Carl-von-Ossietzky-Straße 9-11, 26129 Oldenburg, Germany; 3 Institute for Ophthalmic Research, Centre for Ophthalmology, University of Tübingen, Elfriede-Aulhorn-Straße 7, 72076 Tübingen, Germany; 4 Research Center Neurosensory Science, University of Oldenburg, Carl-von-Ossietzky-Straße 9-11, 26129 Oldenburg, Germany

**Keywords:** subretinal injection, gene therapy, U1 snRNA, U6 snRNA, AAV, splice correction, Opa1, ADOA, optic atrophy, therapeutic treatment

## Abstract

Efficacy and safety considerations constitute essential steps during development of in vivo gene therapies. Herein, we evaluated efficacy and safety of splice factor-based treatments to correct mutation-induced splice defects in an *Opa1* mutant mouse line. We applied adeno-associated viruses to the retina. The viruses transduced retinal cells with an engineered U1 snRNA splice factor designed to correct the *Opa1* splice defect. We found the treatment to be efficient in increasing wild-type *Opa1* transcripts. Correspondingly, Opa1 protein levels increased significantly in treated eyes. Measurements of retinal morphology and function did not reveal therapy-related side-effects supporting the short-term safety of the treatment. Alterations of potential off-target genes were not detected. Our data suggest that treatments of splice defects applying engineered U1 snRNAs represent a promising in vivo therapeutic approach. The therapy increased wild-type *Opa1* transcripts and protein levels without detectable morphological, functional or genetic side-effects in the mouse eye. The U1 snRNA-based therapy can be tailored to specific disease gene mutations, hence, raising the possibility of a wider applicability of this promising technology towards treatment of different inherited retinal diseases.

## 1. Introduction

Splicing of nuclear pre-mRNAs is defined by the excision of introns and the re-ligation of exons to generate mature transcripts. It depends on the identification of exonic sequences within the pre-mRNA as well as the recognition of splice sites at the borders between exons and introns [1]. Splice factors have an essential role in this process, including the U1 small nuclear ribonucleoproteins (U1). They are composed of proteins and a small nuclear RNA (snRNA), and mediate splicing by dynamic and orchestrated interactions between pre-mRNAs and splicing relevant proteins [2]. Spliceosome formation is initiated by recruitment of U1 to the splice donor site (SDS), a process that involves Watson-Crick base-pairing of U1 with the first six nucleotides of the 5′ end of the intron (+1 to +6) and the last three nucleotides of the 3′ end of the exon (−3 to −1) [3,4]. In a later step, U6 is recruited to the SDS, as part of a heterotrimeric complex containing U4-U5-U6. While U1 is released from the spliceosome, U6 generates base-pairs with the nucleotides at positions +4 to +6 of the SDS [5,6]. With the completion of splicing, the splice factors are released from the mature transcript and recycled [3].

Splicing defects are the cause of many human diseases. About 10–20% of human mutations affect canonical splice sites and lead to pathogenic splice defects (Human Gene Mutation Database [HGMD], http://www.hgmd.cf.ac.uk/ac/index.php, accessed on 20 March 2022) [7]. It has been demonstrated that aberrant splicing can be caused by mutations that reduce the interaction between the SDS and U1 [8]. To compensate for mutation-induced defects in pre-mRNA splicing, we and others previously engineered U1 to increase complementarity with the mutated SDS. This gene therapeutic approach on transcript level has been employed successfully in vitro and in cell cultures [9,10,11,12,13,14,15,16,17,18]. Furthermore, we previously demonstrated that a combination of engineered U1 and U6 can lead to improved correction of mutation-induced splice defects [12]. Additional studies suggested that U1 can be efficient in other mouse tissues than the retina [19,20,21].

Mutations in the human Optic Atrophy 1 gene (*OPA1*) lead to a progressive degeneration mainly of retinal ganglion cells (RGC) and their axons which project through the retina and into the optic nerve [22]. *OPA1* mutations are associated with autosomal dominant optic atrophy (ADOA; OMIM: 165500) [23,24], and are the major cause of ADAO [25]. Symptoms of ADOA usually involve slow bilateral vision loss with highly variable severity [26,27,28]. In about 20% of cases, *OPA1* mutations may also lead to syndromic ADOAplus characterized by additional extraocular clinical features including myopathy, peripheral neuropathy, ataxia, encephalopathy, and sensorineural hearing loss [29]. These multisystemic manifestations indicate that other cell types in addition to RGCs may be affected by *OPA1* mutations. Effective therapeutic interventions to attenuate or reverse *OPA1*-associated ADOA do not yet exist.

Opa1 mouse models provided valuable tools to analyze the pathomechanisms of *Opa1* mutations and to develop potential treatment options [30,31,32,33]. In this study, we used the *Opa1*:c.1065+5G>A (*Opa1*^enu/+^) mouse line as a model to evaluate treatments of pathogenic splice donor site defects. The splice defect in the mouse line *Opa1*^enu/+^, carrying the splice donor site mutation *Opa1*:c.1065+5G>A, is well characterized [30,34,35,36]. Notably, *Opa1*^enu/+^ is the only published Opa1 mouse model affected by a SDS mutation. The phenotype in the mouse model is a late onset partial RGC degeneration with slow progression detectable in approximate 1.5-year-old heterozygous mice. The extent of RGC degeneration is variable among heterozygous animals. Interestingly, the same splice donor site mutation (*OPA1*:c.1065+5G>A) occurred in human ADOA patients and caused pathogenic transcript alterations that are highly comparable between the mouse retina and patient-derived cells [37].

Herein, we present a proof-of-principle study that aimed to evaluate short-term efficacy and safety of U1-mediated treatments in vivo in the mouse eye. Our study is the first to demonstrate U1-based splice correction and safety in the retina of an in vivo model, an essential prerequisite for further therapy development.

## 2. Materials and Methods

### 2.1. Generation and Cloning of Mutation-Adapted U1/U6 Cassettes

Wild-type human U1 including its endogenous promotor (U1.wt, 614 bp) was amplified from genomic DNA introducing restriction sites for *BcuI* (BcuI_BshTI_U1_fwd: 5′-AGCTACTAGTACCGGTGTTAGCGTACAGTCTACTTTTGA-3′) and *XbaI* (XbaI_U1_rev: 5′-CGCGTCTAGAGTAAGGACCAGCTTCTTTGG-3′). Site-directed mutagenesis was performed on U1.wt template in three steps using Phusion Polymerase (Thermo Fisher, Darmstadt, Germany). Fragment 1 was mutagenized using BcuI_BshTI_U1_fwd and mutagenesis primer 1 (U1_opa1_rev: 5′-ggcccaagatctcatatttacatcgcaggggagatacc-3′). Fragment 2 was mutagenized using mutagenesis primer 2 (U1_opa1_fwd: 5′- ggtatctcccctgcgatgtaaatatgagatcttgggcc-3′) and XbaI_U1_rev. Both fragments were fused by PCR with primers BcuI_BshTI_U1_fwd and XbaI_U1_rev to yield mutation-adapted U1 cassette (U1.ad, 614 bp).

Wild-type human U6 including its endogenous promotor (U6.wt, 936 bp) was amplified from genomic DNA introducing restriction sites for *XbaI* (XbaI_U6_fwd: 5′- CGCGTCTAGACTCGCTAGGGTCACGTCTCTC-3′ and XbaI_U6_rev: 5′- CGCGTCTAGACAGAGGCAAGATGGGAAAGATC-3′). Site-directed mutagenesis and PCR were performed on U6.wt template as described above using primers XbaI_U6_fwd and XbaI_U6_rev with two mutagenesis primers (U6_opa1_fwd: 5′-ctaaaattggaacgatattgagaagattagcatggcccctg-3′ and U6_opa1_rev: 5′- caggggccatgctaatcttctcaatatcgttccaattttag-3′) to yield mutation-adapted U6 cassette (U6.ad, 936 bp). 

The target vector pscAAV2.1_EF1a_GFP_SV40pA was linearized by restriction digestion with *BcuI* and *XbaI* excising its EF-1a, eGFP and poly(A) cassettes in the process. U1.wt and U1.ad cassettes were digested with *BcuI* and *XbaI* and ligated to the linearized pscAAV2 vector using T4 Ligase (Thermo Fisher, Darmstadt, Germany) yielding plasmids pscAAV2_U1.wt and pscAAV2_U1.ad. 

U6.wt and U6.ad cassettes were digested with *XbaI* and ligated to the *XbaI* linearized pscAAV2_U1.wt and pscAAV2_U1.ad plasmids yielding four plasmids: pscAAV2_U1.wt_U6.wt (U1/U6.wt), pscAAV2_U1.ad_U6.wt (U1.ad), pscAAV2_U1.wt_U6.ad (U6.ad, not used in this study) and pscAAV2_U1.ad_U6.ad (U1/U6.ad). All plasmids were verified by sequencing.

### 2.2. Generation and Purification of AAV2/8

Generation of AAV2/8 was performed for each of the three pscAAV2_U1_U6 plasmids (pscAAV2_U1.wt_U6.wt (U1/U6.wt), pscAAV2_U1.ad_U6.wt (U1.ad), and pscAAV2_U1.ad_U6.ad (U1/U6.ad)) as previously described [38]. We used molar ratios of 1:2:1 of the capsid plasmid pLT-RC08, the pHGTI-Adeno1 helper plasmid, and the pscAAV2 plasmid and yielded a total of approx. 500 µL viral suspension each. Virus titer was quantified by qPCR using the linearized pscAAV2 vector with primers binding to the U1 cassette (U1_fwd: 5′-CACATTTGGGGAAATCGCAGG-3′ and U1_rev: 5′-CCGTGTGTGTAAAGAGTGAGG-3′). The AAV2/8 carrying the GFP-expressing plasmid (pssAAV-CMV-eGFP) was generated correspondingly.

### 2.3. Mice and Subretinal Injection

All animal experiments adhered to the ARVO Statement for the Use of Animals in Ophthalmic and Vision Research and were approved by the Niedersächsisches Landesamt für Verbraucherschutz und Lebensmittelsicherheit (LAVES, ref. no. 15-1829).

*C3HeB/FeJ-OPA1c.1065+5G>A* mice were bred to generate heterozygous (*Opa1*^enu/+^) and wild-typic (*Opa1*^+/+^) offspring. In a randomized fashion, young adult mice (between 1 and 4 months old) from both genotypes were injected with AAV2/8 that carried either U1/U6.wt, U1.ad or U1/U6.ad cassettes mixed 5:1 with AAV2/8 carrying GFP-expressing plasmid (pssAAV-CMV-eGFP) in one eye and PBS or AAV2/8-GFP in the other. A volume of 1.5 µL at a titer of 1.8 × 10^11^ viral particles/mL was injected per eye. 

Mice were anaesthetized with a mixture of Fentanyl (0.05 mg/kgbw), Medetomidin (0.5 mg/kgbw) and Midazolam (5 mg/kgbw) intraperitoneally and pupil-dilating eye-drops were applied (5% Neosynephrin and 1% Tropicamid) for 2 minutes. Fundus imaging was carried out prior to injection on a Phoenix Micron III high-resolution retinal imaging microscope (Phoenix Technology Group, Bend, OR, USA). Subretinal injections were performed by the transcorneal approach [39]: The cornea was punctured at the nasal side close to or at the limbus with a 30 G cannula, and a 33 G needle attached to a Hamilton microliter syringe (Hamilton, Reno, NV, USA) was inserted through the hole. While the iris may be punctured by this method, touching the lens can be avoided in a free-hand injection as the angle of the injection needle is steep enough to pass it. When the tip of the needle penetrated the retina a volume of 1.5 µL of viral solution was applied. The needle was removed and optical gel (Visc Ophthal, Berlin, Germany) was applied. Animals were placed on a heating mat and remained anaesthetized for one hour after injection; at that time, the antidote mixture (50 µL of Naloxon [0.12 mg/kgbw], Flumazenil [0.2 mg/kgbw] and Atipamezol [0.75 mg/kgbw]) was applied subcutaneously. 

Mice were anesthetized after 3–4 weeks of therapy and fundus images including GFP-fluorescence were acquired as described above. To verify the therapeutic duration, RNA analyses (see below) were additionally performed after 8 weeks of therapy. Afterward the treatment, cervical dislocation was performed and eyecups were harvested for further analyses. Eyes displaying GFP signals in less than approximately 50% of the fundus area were excluded from any further analyses, except for non-injected or PBS-injected samples.

### 2.4. Analysis of Transcript Levels with Semi-Quantitative RT-PCR

We extracted RNA from full eyecups preparations using the NucleoSpin RNA kit (Macherey-Nagel, Düren, Germany) according to the manufacturer’s manual. Reverse transcription was carried out using SuperScript III reverse transcriptase (Thermo Fisher, Darmstadt, Germany) and 200-500 ng of RNA per sample. A control reaction was performed without reverse transcriptase (-RT).

RT-PCR reactions were performed using HotFire Taq (Solis Biodyne, Tartu, Estonia) with 1 µL of cDNA as template and *Opa1*-specific primers (OPA1_1011_fwd: 5′-GACGACAAAGGCATCCACCA-3′ and OPA1_1510_rev: 5′-GTTTCCTTTGTGTCGGGAGC-3′) binding to exons 7 and 13, respectively, resulting in PCR fragments of 500 bp from the wild-type allele and 419 bp from the mutated allele (exon 10 skipping during splicing resulted in shorter fragments). Relative band intensities were quantified with Bio-Rad ImageLab v6.0 (Bio-Rad, Feldkirchen, Germany) using the “Auto-detect Bands” function and manual alignment. Values were compared between the eye receiving one of the U1-AAV2/8 and the PBS- or mock-injected control eye from the same animal. All RT-PCRs were technically replicated (two separate cDNA syntheses and two PCR analyses each) at least three times and the mean value was used for statistical analyses. We compared values from eyes that received the same therapeutic construct with control eyes resulting in the following group sizes for semi-quantitative RT-PCR analyses: PBS/GFP controls (n = 25), U1.wt_U6.wt (n = 9), U1.ad_U6.wt (n = 13) and U1.ad_U6.ad (n = 9). 

### 2.5. Sanger Sequencing of Vectors and Opa1 Transcripts

PCR fragments were excised from agarose gels and purified using Macherey-Nagel’s NucleoSpin Gel and PCR Clean-up kit (Macherey-Nagel, Düren, Germany) according to the manufacturer’s manual. DNA concentrations were measured with an Eppendorf BioSpectrometer (Eppendorf, Hamburg, Germany) and 10-20 ng of each sample were used for sequencing. Cycle sequencing was performed using BigDye Terminator v3.1 (Thermo Fisher, Darmstadt, Germany) with one of the primers applied in RT-PCR reactions. Alignment and verification with the reference sequence were performed in SnapGene v5.1.3.1 (GSL Biotech, San Diego, CA, USA).

Vectors were sequence verified either with primer BcuI_BshTI_U1_fwd, XbaI_U1_rev, XbaI_U6_fwd, or XbaI_U6_rev. Two vector-specific primers (pscAAV2_3prime_seq_R: 5′-gcaacaggaaaaacgctcatgg-3′ and pscAAV2_ ori_seq_fwd: 5′-gcgtcgatttttgtgatgctcg-3′) were also applied for Sanger sequencing.

### 2.6. Western-Blot Analysis

Retina and RPE were isolated and lysed in RIPA buffer supplemented with protease inhibitors (S8830, Merck, Taufkirchen, Germany). After 5 min incubation on ice, the supernatant was cleared from insoluble debris by centrifugation (10 min, 6000× *g*, 4 °C). 15 µg of protein lysate was loaded per lane, separated on a 10% PAGE, and blotted onto a PVDF-membrane. After blocking with 5% BSA/TBST for 1 h at room temperature, the membrane was incubated over night at 4 °C with an OPA1-specific antibody (1:1000 diluted, mouse anti-OPA1 clone 18, cat. 612607, BD Biosciences, Heidelberg, Germany) in blocking buffer. As loading control, a mouse anti-GAPDH antibody (Merck, Chemicon, MAB374) was used. A peroxidase-conjugated goat anti-mouse antibody (NB7539, Novus) was used as the secondary antibody, followed by ECL detection. Relative protein levels were calculated based on band intensity quantifications using ImageLab software (Bio-Rad, Feldkirchen, Germany).

### 2.7. Histological Analyses of Retinal Slices

Eyecups were prepared after euthanizing the animals. The cornea was pierced with a lancet at the border between cornea and sclera and the whole eye was incubated in 4% paraformaldehyde for 5 minutes. Afterwards, the cornea was cut off along the equator between cornea and sclera and eyecups were incubated for 2 hours in 4% PFA. Post fixation, eyecups were washed with PBS and incubated in PBS with 30% sucrose overnight at 4°C, followed by embedding in Tissue-Tek O.C.T. compound (Sakura Finetek, Torrance, CA, USA) and storage at −20°C until cutting (similar as previously described [38]).

Histological slices of 20 µm were cut from an area around the optic nerve and the middle third of the eyecup on a Thermo Scientific CryoStar NX70 cryotome (Thermo Fisher, Darmstadt, Germany). GFP and DAPI fluorescence was analyzed on an AxioScan Z1 microscope (Zeiss, Oberkochen, Germany) at 5–10× magnification. Adjacent slices from the region including the optic nerve head were used for further analyses.

Outer nuclear layer thickness was measured at two regions, proximal and distal to the optic nerve head, using FIJI software [40]. Analyzed groups included: wild-type/non-injected (n = 6), wild-type/U1.wt-injected (n = 5), wild-type/U1.ad-injected (n = 9), heterozygous/non-injected (n = 5), heterozygous/GFP-injected (n = 9), heterozygous/U1.wt-injected (n = 9) and heterozygous/U1.ad-injected (n = 16). For each n, retinal thickness was measured in 2-3 adjacent slices at both regions and the means were used for comparison between groups.

### 2.8. Electroretinogram (ERG) Measurements

Eyes were grouped by their fundus image into three categories of injection quality. The lowest quality category was excluded from ERG analyses. High and medium categories were analyzed by ERGs. Animals were dark-adapted overnight (>12 h) before ERG recordings. The animals were handled under dim red light (660 nm). Mice were anaesthetized with a mixture of Fentanyl (0.05 mg/kgbw), Medetomidin (0.5 mg/kgbw) and Midazolam (5 mg/kgbw) intraperitoneally and pupil-dilating eye-drops were applied (5% Neosynephrin and 1% Tropicamid) for 2 minutes. Eyes were moistened with a thin layer of ViscOphthal gel (Dr. Winzer Pharma GmbH, Berlin, Germany). Animals of either sex were used and their ages ranged from 3 to 8 months. For the ERG recordings, the experimenter was blinded to the treatment conditions of the eyes. Full-field ERGs were recorded binocularly. Two platinum coil electrodes were placed on the corneal surface of the left and right eye, respectively. A platinum needle reference electrode was inserted subcutaneously on the forehead. Another platinum needle grounding electrode was inserted at the base of the tail. Mice were placed on a custom-built temperature-regulated stage during the recording. Corneal potentials were amplified (500×) and bandpass filtered (0.1–300 Hz) using a differential amplifier (Model 3000, A-M Systems, WA, USA) and digitized at 10 kHz (PowerLab, ADInstruments). A calibrated Ganzfeld ERG stimulation arena was used for light stimulation (Q450, Roland Consult). ISCEV standard intensities were used [41]. For scotopic ERGs, white-light flashes with intensities of 0.0003, 0.0009, 0.003, 0.0095, 0.03, 0.095, 0.3, 0.95, 3, and 9.5 cd·s/m^2^ were used. Each intensity was repeated 10 times. Inter-stimulus intervals were 2 s for intensities 0.0003–0.0095 cd·s/m^2^, 5 s for intensities 0.03–0.3 cd·s/m^2^, 10 s for intensities 0.95–3 cd·s/m^2^, and 20 s for intensity 9.5 cd·s/m^2^, respectively. For photopic flash stimulation, the animals were light-adapted at a background intensity of 25 cd/m^2^ for 600 s. This intensity was also used as a background during the photopic stimulation, which consisted of 10 repetitions each, with intensities of 0.03, 0.095, 0.3, 0.95, 3, and 9.5 cd·s/m^2^. The a-wave amplitude was defined as the voltage minimum after stimulus onset (0–300 ms) relative to the average voltage before stimulus onset (−100–0 ms). The a-wave implicit time was defined as the time of the voltage minimum relative to the stimulus onset. The b-wave peak was defined as the maximum of the low-pass filtered (cutoff frequency: 75 Hz) response voltage in a time window of 0–300 ms after stimulus onset. The b-wave amplitude was measured from the trough of the a-wave to the b-wave peak. The b-wave implicit time was defined as the time of the voltage maximum relative to the stimulus onset. The a- and b-wave amplitudes and implicit times were visually inspected and manually corrected if necessary. In addition, a photopic flicker ERG was measured over a range of frequencies (2, 4, 8, 16, 32 Hz) at an intensity of 3 cd·s/m^2^. For a given intensity and frequency, responses were averaged across at least 10 stimulus periods and the amplitude was defined as the difference between the maximum and the minimum mean response.

### 2.9. Off-Target Gene Analyses

Potential off-target sites for mutation-adapted U1 were identified by bioinformatic searches for full binding sequence (GATGTAAAT) in coding regions of the mouse genome (mm39) and filtering by the following criteria: target sequence located between 50 and 200 bp downstream of the beginning of a coding exon (potentially shortening the exon if recognized as a bona fide donor site) or between 1 and 60 bp downstream of the beginning of an intron (potential for partial intron retention). Genes containing sites fitting these criteria were checked for expression in the visual system and association with retinal phenotypes on Mouse Gene Expression Database (http://www.informatics.jax.org/expression.shtml, accessed on 18 February 2021). For all of these genes with potential off-target effects, primers spanning the exon/intron region of the target sites were designed (Supplementary Table S1). RT-PCR and subsequent Sanger sequencing were performed on cDNA comparing injected and control eyes (n = 5 animals).

### 2.10. Statistical Analysis

Experiments were replicated at least three times unless otherwise specified. Data are presented as mean ± standard deviation (SD). Error bars indicate the SD. Statistical significance was calculated using Mann-Whitney U test of the R software package (version 3.6) unless otherwise specified (***: *p* < 0.001; **: *p* < 0.01; *: *p* < 0.05; n.s.: not significant, *p* > 0.05).

## 3. Results

### 3.1. Engineered U1 Treatment Rescues Opa1 Splice Defect

**Figure 1:**
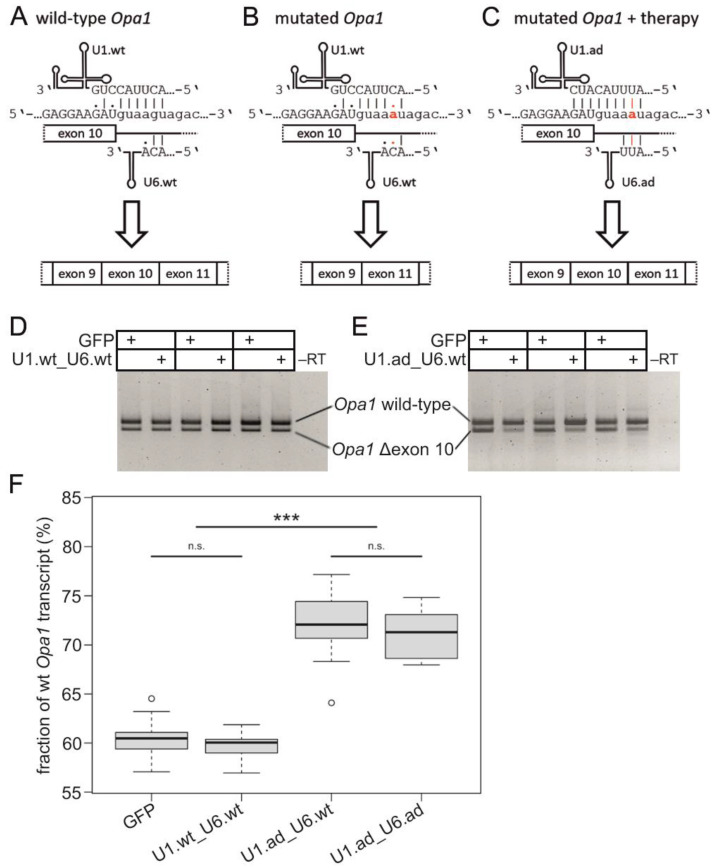
Correcting *Opa1* splice defects with engineered U1 splice factors. (**A**) Wild-type U1 (U1.wt) binds with seven Watson-Crick base-pairings to the *Opa1* wild-type splice donor site of exon 10. Wild-type U6 (U6.wt) binds with only two base-pairings to the *Opa1* pre-mRNA. (**B**) The mutation c.1065+5G>A abolishes Watson-Crick base-pairing at the highly-conserved +5 position in the intron (highlighted in red), leading to skipping of exon 10 during splicing. (**C**) Our treatment strategy uses engineered U1 (U1.ad) to overcome the effect of the mutation c.1065+5G>A and to bind to all nine base pairings of the mutated splice donor site of exon 10 in *Opa1*. Engineered U6 (U6.ad) can bind with all three possible base-pairings. (**D**,**E**) RT-PCR analyses of *Opa1* splicing after treatment with engineered U1 in three *Opa1^enu/+^* mice each. The larger RT-PCR fragment corresponds to the correctly spliced wild-type *Opa1*, the shorter corresponds to *Opa1* skipping exon 10. (**D**) The U1.wt_U6.wt treatment did not change *Opa1* splicing in *Opa1^enu/+^* eyes compared to the contralateral mock (GFP) controls. (**E**) The fraction of correctly spliced wild-type *Opa1* transcripts was increased upon treatment with U1.ad_U6.wt compared to the contralateral mock control. (**F**) Quantification of *Opa1* wild-type and exon10 skipping fragments revealed a significant increase in relative wild-type band intensity (with respect to total *Opa1* transcripts) upon treatment with U1.ad. Statistical significances were calculated by Mann-Whitney U test. ***: *p* < 0.001; n.s.: not significant, *p* > 0.05. –RT: cDNA reaction without reverse transcriptase.

The *Opa1^enu/+^* mouse model carries an *Opa1*:c.1065+5G>A SDS mutation that causes a splice defect leading to exon 10 skipping and thus an in-frame deletion of 81 nucleotides (Figure 1A,B) [30]. The mutation interferes with the base-pairing between the *Opa1* SDS and the wild-type splice factors U1 and U6 (U1.wt and U6.wt; Figure 1A,B). To restore correct splicing of *Opa1* exon 10, we generated engineered U1 and U6 that show full base pair complementarity to the mutated SDS of exon 10 (U1.ad and U6.ad; Figure 1C). We produced AAV2/8 vectors expressing different combinations of the engineered or wild-type U1 and U6: U1.wt_U6.wt, U1.ad_U6.wt, and U1.ad_U6.ad. 

After treatment, we extracted total RNAs from whole eyecups and quantified *Opa1* transcripts that either included or excluded exon 10 (Figure 1D,E). Sanger sequencing of RT-PCR products confirmed the identity of PCR fragments (Supplementary Figure S1). The ratio between wild-type *Opa1* and mutant *Opa1* transcripts skipping exon 10 was not altered in U1.wt_U6.wt injected eyes compared to contralateral mock injected eyes (Figure 1D). In contrast, injections of U1.ad_U6.wt or U1.ad_U6.ad showed therapeutic efficacy and increased the fraction of wild-type *Opa1* transcripts at the expense of mutated *Opa1* (Figure 1E and Supplementary Figure S2, respectively). Quantification of the relative RT-PCR band intensities from treated eyecups confirmed that U1.ad_U6.wt injections significantly increased the wild-type *Opa1* transcripts, while reducing mutated *Opa1* transcripts (U1.ad_U6.wt: 72.0 ± 3.4% (n = 13); U1.wt_U6.wt: 59.6 ± 1.7% (n = 9); PBS: 60.6 ± 1.9% (n = 25)) (Figure 1F). Similarly, a significant increase in *Opa1* wild-type transcripts was detected in eyes treated with U1.ad_U6.ad (71.2 ± 2.4% (n = 9)) (Figure 1F). Differences in *Opa1* splicing between U1.wt_U6.wt and mock injected eyes were not detected (Figure 1F). Notably, no significant differences between the two treatments, either U1.ad_U6.wt or U1.ad_U6.ad, were revealed (Figure 1F), indicating that the co-expression of engineered U1 and U6 did not improve the efficacy of *Opa1* exon 10 inclusion to a detectable level. Consequently, we decided to continue our studies with U1.ad_U6.wt treatments only.

Taken together, our results show a significant increase of wild-type *Opa1* transcript expression in the eye upon AAV2/8-mediated retinal treatment with engineered U1 splice factors. We did not observe unwanted splice-altering effects in *Opa1* transcripts after treatment with either wild-type or engineered U1/U6.

### 3.2. Opa1 Protein Levels Are Increased upon U1 Therapy

**Figure 2:**
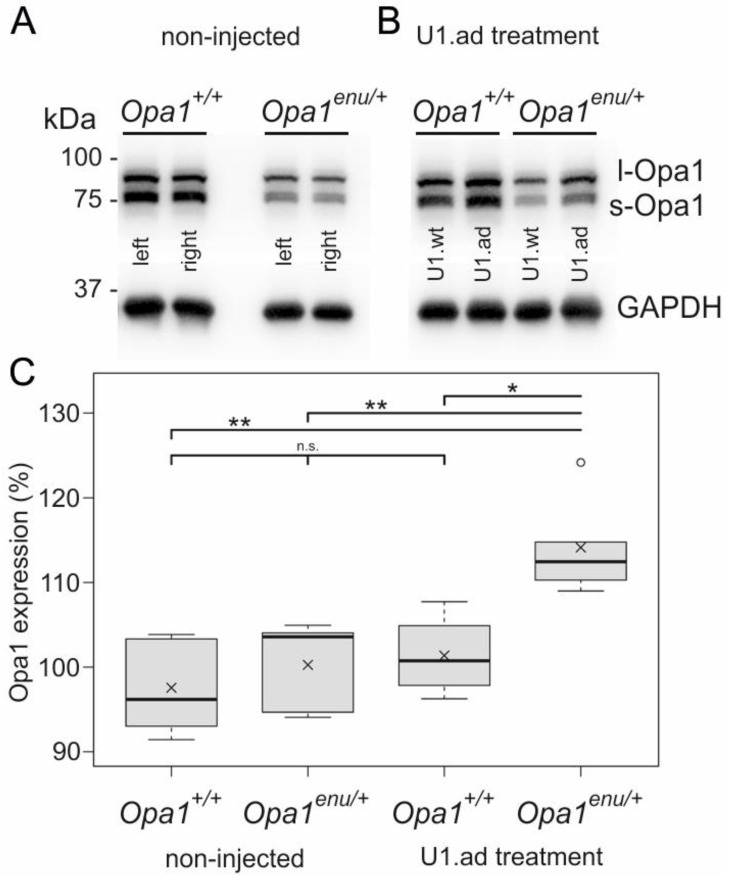
Treatment with engineered U1 specifically increased Opa1 protein expression in *Opa1*^enu/+^ mutant mouse eyes. (**A**,**B**) Western blot analyses of Opa1 protein expression in total homogenates of retina and RPE. Left and right eyes of each animal are shown next to each other. GAPDH was used as a loading control. (**A**) In non-injected animals, Opa1 protein expression was reduced in *Opa1^enu/+^* eyes compared to wild-type eyes. (**B**) Opa1 protein levels were not changed in wild-type eyes upon U1 treatment (left). In *Opa1^enu/+^* eyes injected with engineered U1 (U1.ad), Opa1 protein levels were increased compared to the contralateral control eye injected with U1.wt (right). (**C**) *Opa1^enu/+^* eyes, but not wild-type eyes, treated with engineered U1.ad showed a significant increase of Opa1 protein relative to control (U1.wt) treated contralateral eyes. Quantification of Western blots was performed by comparing Opa1 protein expression of one eye (set to 100% as internal control) to the contralateral eye of the same animal. Statistical significances were calculated by Mann-Whitney U tests. ** *p* < 0.01; * *p* < 0.05; n.s., not significant, *p* > 0.05.

Next, we compared Opa1 protein expression in total homogenates of untreated heterozygous mutant (*Opa1^enu/+^*) and wild-type (*Opa1^+/+^*) mouse eyes. Untreated *Opa1^enu/+^* mice exhibited clearly reduced Opa1 levels compared to *Opa1^+/+^* mice (Figure 2A). Left and right eyes from the same mouse showed highly similar Opa1 protein levels. Our findings confirmed previous observations: Protein levels of Opa1 were reduced by appr. 50% in mouse models and human fibroblasts carrying heterozygous *Opa1* mutations [30,37]. 

To further evaluate the therapeutic potential of engineered U1, we asked whether the increase in wild-type *Opa1* transcripts after U1 therapy translates into elevated Opa1 protein expression. To exclude inter-individual variability, we performed different injections into each of the two eyes of the same animals. *Opa1^enu/+^* and *Opa1^+/+^* mice were injected with U1.ad in one eye, whereas the contralateral eye received U1.wt as control. We compared the treatment effects by measuring Opa1 protein expression in Western blots. While there was no detectable difference between the injected eyes in wild-type mice (Figure 2B, left), treatment with engineered U1 increased Opa1 protein expression in mutant *Opa1^enu/+^* mice (Figure 2B, right). 

Quantification of Western blots was performed by comparing Opa1 expression of one eye (set to 100% as internal control) with the contralateral eye of the same animal. As expected, non-injected animals showed no significant difference in Opa1 protein levels between left and right eyes in both, *Opa1^enu/+^* and *Opa1^+/+^* mice (Figure 2A,C(left)). For injected animals, Opa1 levels in the U1.wt treated eye were set to 100% and compared to the contralateral U1.ad treated eye (see Figure 2B,C(right)). Our quantification showed that U1.ad treated *Opa1^enu/+^* eyes had significantly increased Opa1 protein levels (114.1 ± 5.4%, n = 5, normalized to the contralateral control-eyes) (Figure 2C), consistent with the increase of wild-type *Opa1* transcripts (Figure 1E,F). The effect was specific for *Opa1^enu/+^* eyes. Opa1 protein levels in wild-type eyes were not affected by injection of engineered or wild-type U1 (101.4 ± 4.2%, n = 4). 

In summary, the in vivo treatment of the retina with engineered U1 in *Opa1^enu/+^* mutant eyes concomitantly increased *Opa1* transcript and protein levels. Our results indicate efficacy of the splice correction therapy on the molecular level. 

### 3.3. Off-Target Splicing Is Not Affected by the U1 Treatment

**Figure 3:**
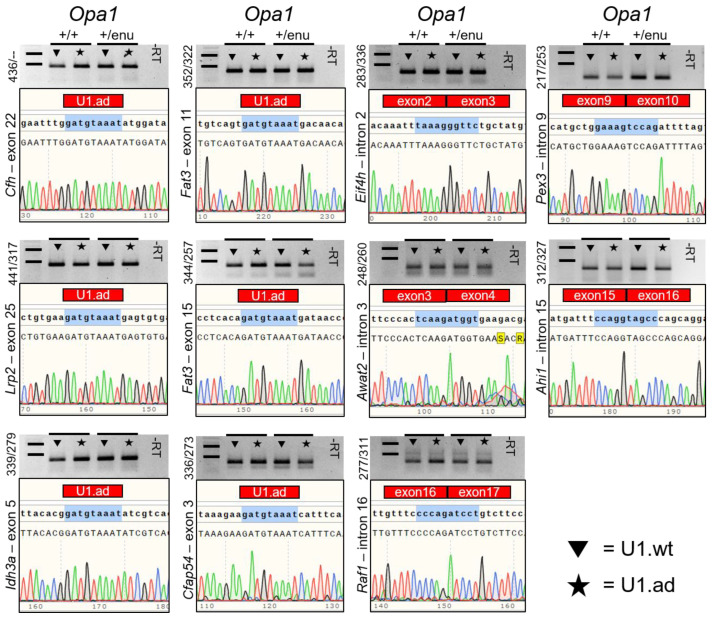
Off-target analyses of engineered U1 on pre-mRNA splicing. Potential off-target transcripts from 11 genes previously associated with retinal phenotypes were analyzed by RT-PCR using exon/intron-spanning primers. Off-target effects on splicing were not detected. Additional bands, indicating splice alterations, were not identified in any of the 11 transcripts upon U1-based treatments, neither in wild-type (*Opa1^+/+^*, n = 5) nor in heterozygous (*Opa1^enu/+^*, n = 5) mice. Sequencing of the RT-PCR fragments confirmed correct splicing of the potential off-target genes. U1.ad marks potential binding sites of engineered U1. Exon to exon borders are highlighted. Size marker represent 500 and 1000 base pairs (bp). Numbers to the left of each gel image indicate the expected wild-type fragment sizes (first number) as well as sizes of potential off-target splicing events (second number).

To analyze the RNA safety profile of the U1 treatment, we verified whether splicing alterations in potential off-target genes occurred following AAV2/8-U1 transduction of the retina. We analyzed genes previously associated with diseases of the visual system and bioinformatically identified potential binding sites of the engineered U1 (GATGTAAAT). This procedure predicted 57 or 37 binding sites with the potential to result in exon-truncation or intron-retention, respectively. From these two sets of binding sites, eleven U1 binding sites occurred in genes that have previously been associated with visual phenotypes (Supplementary Table S1). We analyzed the corresponding transcripts for splice alternating events and compared treated and control eyecups using RT-PCR (Supplementary Table S1). We did not detect alterations in either fragment sizes or intensities, and we did not find an association with the engineered U1 therapy (n = 5) (Figure 3). Sequencing the PCR products confirmed the wild-type sequences of the transcripts and did not identify sequence alterations in potential off-target transcripts (Figure 3). Despite several attempts, we were unable to establish RT-PCRs amplifying *Ankrd35*.

Overall, expression of engineered U1 did not induce detectable mis-splicing in visual system-associated transcripts. These results support the belief that splice defects in off-target transcripts are not an obvious side effect of U1 treatments.

### 3.4. Retinal Morphology Remains Inconspicuous after Subretinal AAV2/8 Injection

**Figure 4:**
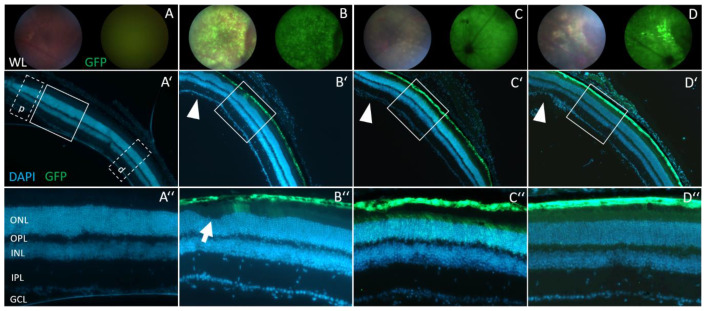
Fundus images and histological morphology of injected eyes. (**A**–**D**) Fundus images of injected eyes after white-light (WL, left) or UV-light with GFP channel detection (right). (**A**) PBS- and non-injected animals only showed background signal. (**B**) GFP fluorescence appeared across the wide areas of the fundus in mice injected with GFP-expressing AAV2/8. (**C**) Wild-type U1 and (**D**) engineered U1 four weeks after subretinal injection. Fundus pictures documented wide-spread GFP-expression and successful transduction of retinal cells. (**A’**–**D’’**) Retinal cryosections document AAV2/8-mediated transductions mainly to the retinal pigment epithelium and photoreceptor layers. ONL thickness was measured at regions marked by boxes (dotted lines) and labelled by p and d in **A’**. At and around the point of injection (arrowheads), mechanical damage to the retina can be detected (as in **D’**). (**A’’**–**D’’**) Magnification of boxes showed that the overall retinal morphology was similar between eyes regardless of the injected construct. Retinal detachments (arrow in **B’’**) were observed occasionally. Panels A, C, D: *Opa1*^+/+^; B: *Opa1*^enu/+^. WL, white light; ONL, outer nuclear layer; OPL, outer plexiform layer; INL, inner nuclear layer; IPL, inner plexiform layer; GCL, ganglion cell layer.

To monitor in vivo retinal morphology and transduction efficacy of AAV2/8 after subretinal therapy, we employed fundus imaging and collected full eyecups from sacrificed injected animals. Between three and eight weeks after injection, GFP signals were detectable across wide areas of the retina in fundus images of injected animals (Figure 4A–D). This observation was confirmed in retinal slices, mainly showing GFP signals in the retinal pigment epithelium (RPE) and photoreceptor layer (Figure 4A’–D’). The photoreceptor layer was widely transduced, but appeared to express less GFP than RPE cells (Figure 4C’’–D’’). These observations are in line with previously published data of transduction efficacies of the AAV2/8 serotype [38,42,43]. Taken together, our results show a successful and wide-spread transduction of outer retinal layers following a single subretinal injection of AAV2/8 viral particles that expressed engineered U1.

To elucidate potential side-effects of treatment with engineered U1 in the retina, we analyzed the retinal layer morphology. Compared to non-injected eyes (*Opa1^enu/+^*: n = 4, *Opa1^+/+^*: n = 8), retinal slices from the area around the optic nerve head showed inconspicuous morphology after injection of AAV2/8 expressing engineered U1 (*Opa1^enu/+^*: n = 14, *Opa1^+/+^*: n = 12), or wild-type U1 (*Opa1^enu/+^*: n = 8, *Opa1^+/+^*: n = 10), or GFP/PBS (*Opa1^enu/+^*: n = 19, *Opa1^+/+^* : n = 12) (Figure 4A’–D’’). At the site of injection, the retina was mechanically damaged by the injection needle (Figure 4D’). While physical disruption of the retinal tissue at the injection site can be considered a side-effect, the retinae tolerated detachment/re-attachment during the course of therapy and showed no detectable morphological differences caused by our therapeutic intervention. 

To detect potential degeneration of retinal cells, we quantified retinal thickness near the site of injection (Table 1). Measurements of outer nuclear layer (ONL) thickness in retinal slices at two regions, proximal and distal to the optic nerve head (see boxes p and d in Figure 4A’), revealed no significant difference (Supplementary Table S2). Thus, alterations of the retinal morphology, e.g., a thinning or swelling of the retina, were undetectable between injected and non-injected *Opa1^enu/+^* animals (Table 1). 

Taken together, we did not detect any significant differences in ONL thickness, neither in proximal nor distal retinal areas of the optic nerve head. Comparing retinae between *Opa1^+/+^* and *Opa1^enu/+^* animals and different treatments resulted in highly similar values. These results indicated the retinal morphology was not affected by the AAV2/8-mediated gene therapy applying engineered U1 splice factors.

### 3.5. Electroretinogram Responses Are Unaltered after U1 Treatments

**Figure 5:**
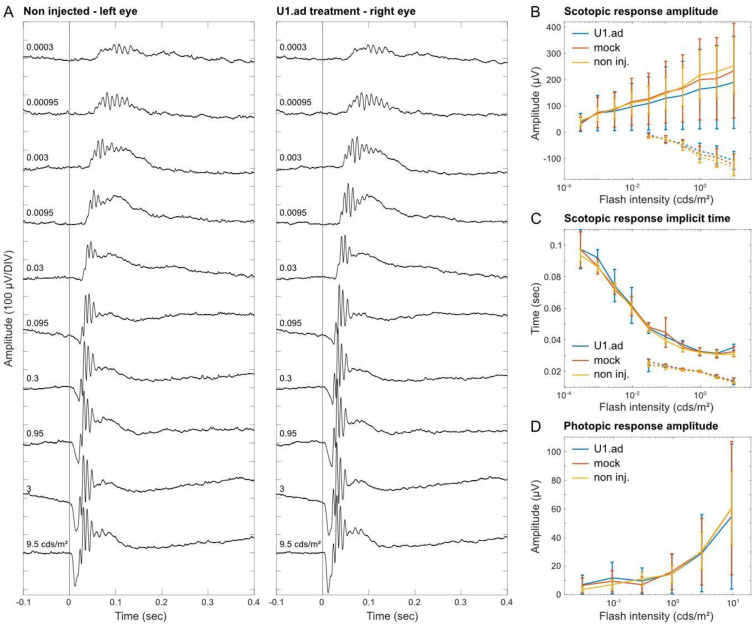
U1-mediated side effects on retinal function were not detected by electroretinography. (**A**) Representative scotopic ERG responses to light flashes of increasing intensity. The left eye was untreated. The right eye was injected with engineered U1 (U1.ad). The ERGs of both eyes were recorded simultaneously. (**B**) Mean scotopic ERG amplitudes. Error bars represent 95% confidence intervals (U1.ad: n = 7, mock: n = 9, non-injected: n = 24). A-wave dashed line, b-wave solid line. (**C**) Scotopic ERG implicit times corresponding to (**B**). (**D**) Photopic ERG amplitudes of light-adapted mice with stimulus flash of increasing intensities corresponding to (**B**).

To verify retinal function and to explore potential side-effects of the U1 treatment, we performed Ganzfeld ERG measurements under photopic and scotopic conditions and compared U1-treated, mock-injected and control eyes. Following the ERG measurement, histological analyses of all analyzed eyes detected only minor mechanical damages caused by the injection procedures and confirmed wide-spread GFP signals across the retinae. Amplitude, waveform, and timing of the a-wave—reflecting the activity of the photoreceptors—were unaltered between treated and control eyes (Figure 5A–D). Furthermore, the b-wave, which reflects excitation and activity of bipolar cells in the inner retina, showed no significant differences across treatment conditions (Figure 5A–D). Groups tested were: injected with U1.ad_U6.wt (n = 7); mock-injected (n = 9); and non-injected (n = 24) (Figure 5B–D). The flicker ERG showed no significant difference between the therapeutic construct, control construct, and the non-injected groups, with overlapping confidence intervals (Supplementary Figure S3).

Overall, our data supported that AAV2/8-mediated U1 treatments of retinal diseases may be considered an efficient and safe gene therapeutic approach that lacks obvious side-effects towards retinal functionality, morphology and splicing.

## 4. Discussion

We evaluated the efficacy and safety of engineered U1 as a gene therapeutic agent in the retina. We made use of an ADOA mouse model carrying a splice-site mutation in the *Opa1* gene and show that subretinal delivery of engineered U1 via AAV2/8 leads to transduction of the RPE and photoreceptor layers. Following the U1 treatment, we detected increased wild-type *Opa1* expression on the transcript and protein level. Short-term side-effects of the U1-therapy were not detected, especially not on functional level (ERG). The results presented herein suggested that this therapeutic approach might be considered for further evaluation in RGCs, conceivably even in human clinical trials.

The *Opa1*^enu/+^ mouse model is well-suited to evaluate treatments of splice defects, i.e., the *Opa1*^enu/+^ mutation leads to exon skipping [30], one of the most frequently observed mutation-induced splice defects. However, the *Opa1*^enu/+^-mouse model is less suitable to evaluate short-term safety and efficacy of RGC-specific treatments; variability and late onset as well as slow progression of RGC degeneration imply obstacles to short-term phenotypic evaluations [30]. Thus, we did not target RGCs, the most affected cell type in ADOA, but RPE and photoreceptor cells, the most abundant and highly vulnerable cell types in the retina. Their high abundance allowed us to analyze short-term efficacy and safety of the U1-mediated treatment in vivo, with the clear advantage of circumventing more emulated in vitro measurements with enriched and cultivated cells types from the retina. Safety and efficacy of the U1 treatment had not been analyzed in the in vivo retina before. The analyzed cell types enable well-established, highly-sensitive molecular (Figure 3), morphological (Figure 4 and Table 1), and physiological measurements (Figure 5) likely to detect potential short-term side-effects that might be associated with therapeutic interventions. 

Efficient and sustained transduction of RPE and photoreceptor layers was previously achieved after subretinal AAV2/8 injections [42,43,44,45]. Therapeutic effects were transferred to the retina using subretinal injection of AAV2/8, e.g., inhibiting neovascularization [46]. Even more significant, subretinal injections were applied in approved gene therapies to treat retinal diseases in human, e.g., for RPE65 deficiencies [47,48,49]. Delivery routes of gene therapies were only accompanied by minor side effects [50], further supporting the enormous potential of genetic treatments in the retina. 

Our present study was designed as a proof-of-concept at an early stage of retinal therapy. Hence, we did not investigate long-term therapeutic effects nor did we aim at attenuating or reversing the pathogenesis of ADOA. While we were able to show that the U1 therapy increases Opa1 expression on the molecular level—the precondition for any functional therapy—we did not address if the U1 treatment rescues mitochondrial functions or attenuates RGC degeneration on the cellular level; this will require future long-term studies. For isolated RGCs from *Opa1*^Q285STOP^ mice, mitochondrial functions were shown to be compromised [51]. It will be interesting to evaluate whether similar effects can be observed in *Opa1*^enu/+^ derived RGCs and whether they can be rescued applying the U1 therapy.

Haploinsufficiency is the major disease mechanism underlying *OPA1* mutations, i.e., the disease is caused by the loss of gene dose from a single allele that carries a heterozygous mutation [52]. Engineered U1 resulted in a significant upregulation of correctly spliced *Opa1* transcripts from the mutated allele. Given the loss or very mild disease expression frequently observed in *OPA1*-linked ADOA [53,54], it is likely that the increased expression levels of Opa1 will attenuate or even stop the disease progression. Importantly, about 10–20% of all mutations affect splicing, an observation that is almost independent from the affected gene and thus, raises the chance to apply U1 therapies to many different genetic diseases [55]. 

We applied engineered U1 which showed increased binding capacities to the mutated SDS in order to recruit downstream splicing factors and to initiate normal splicing despite of the mutation [2,56,57]. The U1 therapy targets pre-mRNA transcripts derived specifically from the mutated allele and shows the capacity to restore expression levels and isoform ratios of the targeted gene within physiological ranges. In contrast, gene replacement/augmentation strategies typically overexpress a single protein isoform along with limited capacities in restoring physiological expression ratio among isoforms. Notably, viral vectors provide restricted insert sizes—a further limitation to gene augmentation therapies of large genes. In contrast, U1 expression cassettes are only a few hundred base pairs in size and can easily be applied using all viral vectors. The natural chromosomal context of the affected gene and hence regulation of gene expression is maintained, i.e., the endogenous promotor, pre-mRNA processing, and ratios of splice isoforms are not affected. 

*Opa1* is a ubiquitously expressed gene in which different isoforms are relevant to its multiple mitochondrial functions [29,58,59]. Mis-regulated expression of single isoforms or transcripts may thus counteract therapeutic effects. For the complete fusion of mitochondrial inner membranes both, the short and long isoform of Opa1 are required at stoichiometric levels [60,61,62], whereas an excess of the short isoform inhibits fusion [62]. These findings suggested that gene augmentation therapies for *OPA1* may have limited therapeutic efficacy. However, studies in a different *Opa1* mutant mouse line (*Opa1*^delTTAG^) showed some promising therapeutic efficacy by overexpression of a single wild-type *Opa1* isoform including the restoration of visual function and preventing RGC loss [32]. In mice with rotenone induced mitochondrial dysfunction, gene augmentation using single *Opa1* isoforms protected spatial visual function [63]. However, a thorough safety study on gene augmentation therapy for *OPA1* has not been conducted. Regardless, U1-based therapies may provide attractive alternatives to gene augmentation approaches.

While subretinal injections mechanically disrupts the retina at the point of injection, reversible retinal detachment after application of the viral suspension was observed. The viral therapeutic agent did not lead to obvious side effects on retinal morphology. Our findings are in line with other studies showing ONL thickness in the range of approx. 54 µm to 57 µm in retinae of C57BL/6 mice [64,65]. In future studies, an intravitreal injection method and different AAV serotypes might be favorable to minimize mechanical stress to the retina and to specifically target RGCs [38,66]. However, the inner limiting membrane might reduce efficient transduction of retinal cells and transduction is mainly limited to retinal cells facing the vitreous [67]. Compared to subretinal injections, intravitreal injections exhibit a higher risk for intraocular inflammation and humoral immune response [68]. 

Mutations in the 5′ SDS binding region of U1 snRNA, i.e., r.3A>G and r.3A>C that pair with the +6 position of the splice donor site, have been associated with specific forms of cancer [69,70], highlighting the need for careful safety evaluation for therapies based on engineered U1. Importantly, the critical r.3A nucleotide has not been modified in our engineered U1 (see Figure 1A–C) and we did not observe any indication for cancerous cell alterations in the treated retinae. Furthermore, our data did not indicate off-target effects of the treatment on the molecular level, i.e., mis-splicing events caused by the U1 treatment. Apart from RNA and morphological analyses, we also tested for potential side-effects of the U1-based treatment on retinal functionality. ERG measurements of eyes injected with engineered U1 showed no significant differences in parameters representing outer retina function (including photoreceptors and bipolar cells). Although our molecular, morphological and functional analyses of the retina did not suggest splice defects in off-target transcripts, we cannot completely exclude this possibility; future studies might include transcriptome analyses to further address this issue.

Together, we showed for the first time that retinal applications of U1-based gene therapies lead to promising treatment profiles, providing support for further development towards splice donor site treatments of many different retinal diseases. Our findings strongly support that the U1 therapy, as presented herein, did not alter physiological function of the retina. Engineered U1 delivered via AAV2/8 to eyes of *Opa1^enu/+^* mice led to a significant increase in *Opa1* wild-type expression while showing no indications for short-term side-effects on splicing, retinal morphology, and retinal physiology. Future studies will verify these findings in RGCs, investigate long-term safety and efficacy of treatments, and will aim to further evaluate the potential of this promising therapeutic approach towards clinical trials. 

## Figures and Tables

**Table 1:** ONL thickness of the central retina is not altered by the U1 treatments. The average outer nuclear layer (ONL) thickness in micro-meters (µm) is listed with standard deviations. Retinae from *Opa1*^+/+^ (wild-type) and *Opa1*^enu/+^ (heterozygous mutant) animals are compared. ONL thickness was measured at regions proximal and distal to the optic nerve head. Pairwise comparison of ONL thickness at proximal and distal regions between groups using Mann-Whitney U test with Bonferroni correction did not yield significant differences (Supplementary Table S2). The number of biological replicates is indicated in brackets (n = 5–16).

	*Opa1*^+/+^ ONL Thickness (µm)		*Opa1*^enu/+^ ONL Thickness (µm)	
	Proximal	Distal	Proximal	Distal
**non-injected**	56.05 ± 1.21 (n = 6)	52.71 ± 2.08 (n = 6)	57.99 ± 1.24 (n = 5)	56.75 ± 1.10 (n = 5)
**GFP**	-	-	56.08 ± 1.80 (n = 9)	53.97 ± 2.12 (n = 9)
**U1.wt**	56.77 ± 1.57 (n = 5)	52.84 ± 2.35 (n = 5)	56.28 ± 2.73 (n = 9)	53.75 ± 2.72 (n = 9)
**U1.ad**	54.77 ± 3.86 (n = 9)	53.25 ± 3.07 (n = 9)	56.08 ± 2.90 (n = 16)	53.24 ± 2.29 (n = 16)

## Data Availability

The data presented in this study are available on request from the corresponding author.

## Supplementary Materials

The following supporting information can be downloaded at: https://www.mdpi.com/article/10.3390/cells12060955/s1, Figure S1: Sequence verification of *Opa1* wild-type and exon 10 skipping PCR fragments; Figure S2: RT-PCR analyses of *Opa1* splicing after treatment with engineered U1 and U6 in three *Opa1^enu/+^* mice; Figure S3: U1-mediated side effects on retinal function were not detected by flicker electroretinography; Table S1: Primer sequences for off-target analyses; Table S2: Comparison of ONL thickness in central retinal slices by injected construct.
