# Supplementary material for: In Vivo Efficacy and Safety Evaluations of Therapeutic Splicing Correction Using U1 snRNA in the Mouse Retina
Source: Cells. 2023 Mar 21;12(6):955. doi: 10.3390/cells12060955 (PMC10047704; doi:10.3390/cells12060955)
Supplement: Supplementary file 1 [file cells-12-00955-s001.zip › cells-2203629-supplementary.pdf]

Supplementary material

Supplementary table S1: Primer sequences for off-target analyses. Overview of genes containing potential off-target sites for U1.ad binding. The binding position within each gene is indicated by the exon/intron number containing the potential off-target site. Sequences of primers used for RT-PCR amplification are listed from 5´ to 3´. The PCR product sizes for the wild-type and a potentially mis-spliced transcript are given in base pairs (bp).

**gene**	**potential off-target U1 binding site**	**forward primer**	**reverse primer**	**RT-PCR fragment size (bp)**<br>of the wild-type product	**RT-PCR fragment size (bp)**<br>of the predicted off-target product
Ankrd35	exon 14	ctgtgtgccctcaggg	ccatctgttttctgctttattttcc	229	no band
CFap54	exon 3	ctgccgtccaactattacaacg	ctgcatgatgagccgaaagg	336	273
Cfh	exon 22	gggaaatgtgggcctcc	gtatattagtcacatgcatgtgcc	436	no band
Fat3	exon 11	ccaccaggggaggtgg	ccttggcactgaccttcagg	352	322
Fat3	exon 15	cttacctcatccggtctgg	gtccctatctccacccac	344	257
Idh3a	exon 5	ggaccaggaggaaagtgg	ctcgaaggcaaactctgc	339	279
Lrp2	exon 25	gccttttcactgtcctagctc	gtgtgggcagtgatattgtcc	441	317
Ahi1	intron 15	cgccgtgcgggactc	gcagcttagggcaggtgc	312	327
Awat2	intron 3	cttactggcctgtcaccg	gcctgttgtgtcagtggc	248	260
Eif4h	intron 2	cctacgacgatcgggcc	gtcagagcctccttcaggg	283	336
Pex3	intron 9	gtccaggtcttcactgtgcc	gggtcgaaagaattcggcc	217	253
Raf1	intron 16	gctgatggctggggagc	ctcagtgtgagctgcccg	277	311

Supplementary table S2: Comparison of ONL thickness in central retinal slices by injected construct. p-values of the comparison of ONL thickness at proximal (white text on dark grey background) and distal (black text on light grey background) regions between groups ($Opa1^{+/+}$: white background, $Opa1^{enu/+}$: black background) using Mann-Whitney U test with Bonferroni correction (corrected p-values are shown).

		**non**	**U1.wt**	**U1.ad**	**non**	**GFP**	**U1.wt**	**U1.ad**
**inj. construct**	**non**	-	1	1	0.091	1	1	1
	**U1.wt**	1	-	1	0.667	1	1	1
	**U1.ad**	1	1	-	0.252	1	1	1
	**non**	0.64	1	1	-	0.252	0.608	0.233
	**GFP**	1	1	1	0.88	-	1	1
	**U1.wt**	1	1	1	1	1	-	1
	**U1.ad**	1	1	1	1	1	1	-

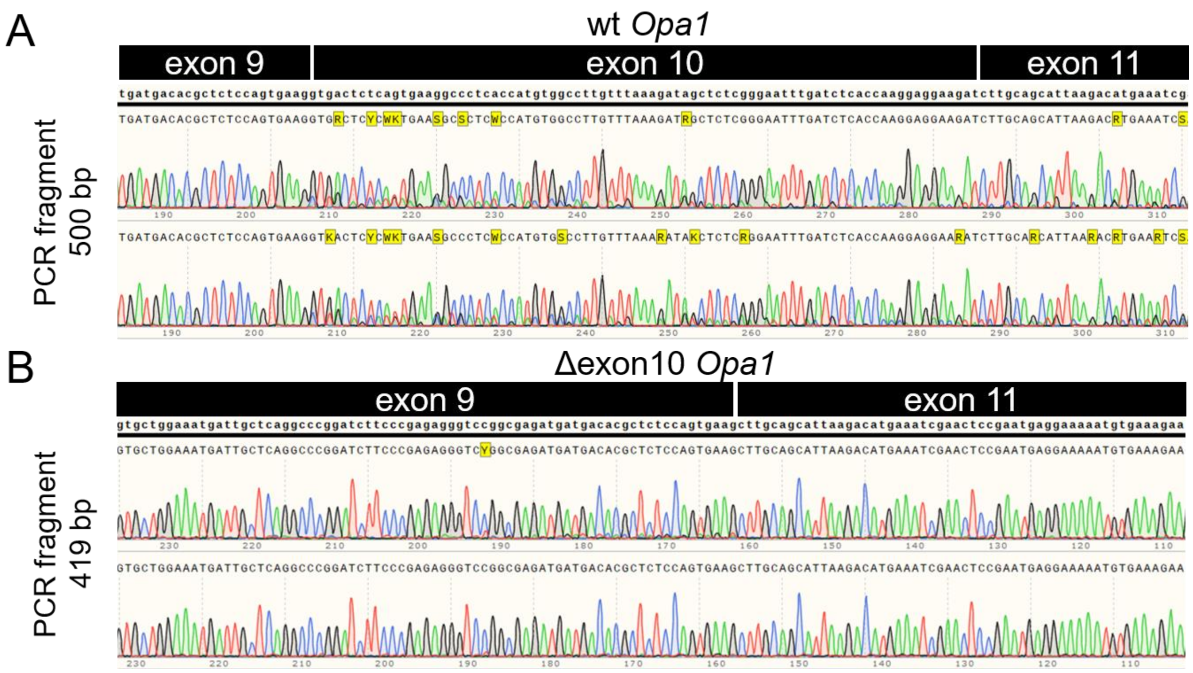

Supplementary Figure S1. Sequence verification of *Opa1* wild-type and exon 10 skipping PCR fragments. (**A**) Sequencing of extracted fragments from RT-PCR gels showed inclusion of *Opa1* exon 10 in the larger fragment (500 bp). (**B**) Skipping of exon 10 during splicing in the smaller fragment (419 bp) was confirmed by Sanger sequencing. Smaller fragments showed the direct transition from exon 9 to exon 11. Notably, sequences from larger fragment showed background base calls in exon 10 which are consistent with a minor contamination by the smaller fragment.

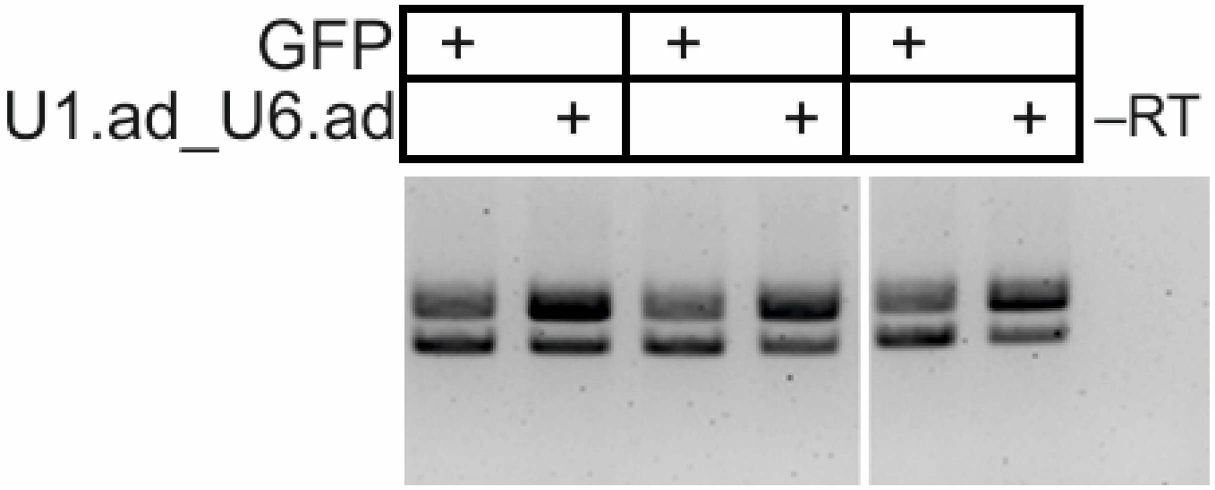

Supplementary Figure S2. RT-PCR analyses of *Opa1* splicing after treatment with engineered U1 and U6 in three *Opa1*$^{enu/+}$ mice. The larger RT-PCR fragment corresponds to the correctly spliced wild-type *Opa1*, the shorter corresponds to *Opa1* transcript skipping exon 10. Correctly spliced wild-type *Opa1* transcripts are increased upon injection of U1.ad_U6.ad compared to the contralateral mock control (for quantification see Figure 1F).

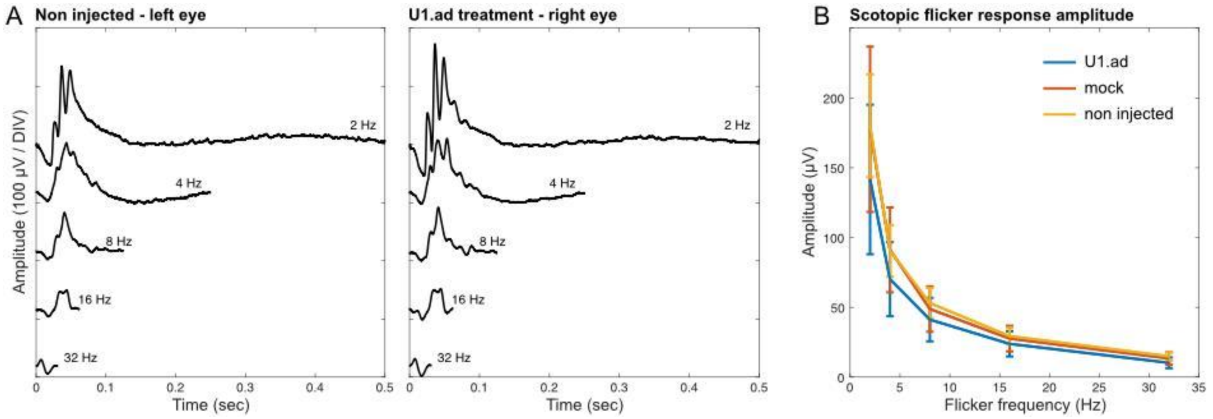

Supplementary Figure S3. U1-mediated side effects on retinal function were not detected by flicker electroretinography. (**A**) Representative scotopic ERG responses to flickering light of varying frequency. The intensity of the light flashes was 3 cd*s/m^2. The left eye was untreated. The right eye was injected with engineered U1 (U1.ad). The ERGs of both eyes were recorded simultaneously. (**B**) Mean scotopic flicker ERG amplitudes. Error bars represent 95% confidence intervals (U1.ad: n=7, mock: n=9, non-injected: n=24).
